# Influence of energy poverty on agricultural water efficiency using a panel data study in China

**DOI:** 10.1038/s41598-023-50971-y

**Published:** 2024-01-24

**Authors:** Hongxu Shi, Yuehua Zhang, Mengyan Bian, Jun Zhang

**Affiliations:** School of Agricultural Economics and Rural Development, Renmin University of China, No. 59 Zhongguancun Ave., Haidian District, Beijing, 100872 People’s Republic of China

**Keywords:** Energy and society, Socioeconomic scenarios

## Abstract

The research attention is increasingly directed towards the effective integration of the 17 United Nations Sustainable Development Goals (SDGs) within the limitations of the real world and amidst intersectoral conflicts. In light of the inextricable relationship between irrigation and energy, the objective of this study is to identify potential avenues for achieving the SDG6 and SDG7 goals of enhancing water use efficiency in agriculture and eradicating energy poverty, respectively. Utilizing data from 30 Chinese provinces from 2002 to 2017, this study explores the dynamic influence of energy poverty on agricultural water efficiency with a system generalized method of moments methodology. The findings suggest that energy poverty may greatly reduce agricultural water efficiency. The heterogeneity study shows that when agricultural water efficiency grows, the negative impacts of energy poverty continue to fade. Based on an assessment of various processes, results suggest that non-farm employment and cropping structure modification is a prominent conduit via which energy poverty negatively influences agricultural water efficiency.

## Introduction

The Sustainable Development Goals were established by the United Nations in 2015. The set of goals include 17 objectives that span various domains, including economic, social, and environmental dimensions. Consequently, the interplay between these goals can give rise to conflicts and intricacies. As an illustration, it advocates for the safeguarding of the environment and ecosystems, concurrently emphasizing the need for job creation, enhanced food production, and poverty elimination. The design of the Sustainable Development Goals (SDGs) demonstrates a shared global commitment to pursuing sustainable development, despite the numerous complexities and obstacles that exist in the real world. As a result, an increasing number of academics and policymakers have directed their attention towards achieving a harmonious equilibrium within various domains and harnessing the potential synergies among diverse sustainable development objectives.

The research contents of this study are situated within the context of Sustainable Development Goal 6 (SDG6) and Sustainable Development Goal 7 (SDG7), and the focus lies on the strategies to improve water usage efficiency in agricultural practices and the elimination of energy poverty are crucial components in achieving SDG 6 and SDG 7. Therefore, the primary objective of this study is to examine the correlation between agricultural water usage efficiency and energy poverty, with the aim of identifying various ways to align these two Sustainable Development Goals (SDGs). In the agricultural sector and rural areas, these two goals are inextricably linked, as there is a strong connection between energy and irrigation^1–6^.

Agriculture is a crucial economic sector in developing nations, but it must contend with inefficient water usage and diminishing water resources. Concerns have been voiced concerning the worldwide scarcity and overexploitation of freshwater resources as a result of economic expansion and rising demand for food, feed, and fuel. Irrigated agriculture consumes around 70 percent of the world's water resources^7^. As industrialization and urbanization continue, there is growing concern about the impact on agricultural water use as water is diverted to other sectors^8^. Simultaneously, demand for food production has been increasing and is projected to rise by around 60 percent by 2050^7^. Furthermore, climate change is expected to worsen the future imbalance between supply and demand for water, as well as increase supply variability^9^. Droughts induced by climate change demand more irrigation and more water to ameliorate, highlighting the significance of improving agricultural water efficiency (AWE). Energy consumption analyses of different agricultural activities demonstrate that irrigation requires far more energy than other agricultural activities^10,11^. Although a number of studies have analyzed the energy efficiency of agricultural production, the essence is to explore the ability of energy to be converted into agricultural output, and these studies do not take into account the fact that farmers often have various troubles with energy use in reality. Hence, it is imperative to investigate the influence of a region’s degree of energy development on agriculture and irrigation. This research perspective diverges from current studies that primarily focus on analyzing direct energy inputs within the agricultural sector.

As the world’s largest developing country, China provides a unique sample for studying the impact of energy poverty on agricultural water efficiency. The following are two reasons for selecting China as the study sample. First, China is still continuing to strengthen its energy facilities and to improve its energy development. Many rural areas in China are still lagging behind in energy construction, and it was not until 2020 that universal access to rural power electricity was fully achieved. If water facilities do not receive sufficient energy support, they are bound to fail to operate efficiently. Energy poverty (EP) is a comprehensive indicator that can effectively characterize the level of energy development in a region^12^. Second, China is not rich in water resources. China’s water availability per capita is about a fourth of the international average^13^. China must improve agricultural water efficiency to achieve agricultural water conservation and food security^14^. In addition, we have seen a number of reports within China of completed wells being unable to irrigate properly due to lack of electricity. Therefore, it is highly likely that the level of energy development in the region has a direct impact on the effectiveness of irrigation facilities, and thus on the efficiency of irrigation. This is one of the important practical contexts for this study to examine energy poverty and agricultural water efficiency. Therefore, China is a suitable sample for studying this element. The results of a study on China would be more reliable and useful. At the same time, the results would be beneficial to other developing countries trying to alleviate water scarcity and energy poverty.

## Materials and methods

All of the data for this study came from publicly available sources, including the China Agriculture Yearbook, China Rural Statistical Yearbook, China Agricultural Machinery Industry Yearbook, China Statistical Yearbook, and China Population and Employment Statistics Yearbook. These data sources are complied into the Express Professional Superior Data Platform, which integrates rich numerical data resources and powerful analytical forecasting system. Details about the data source and variables used in the statistical analyses are presented in the Table A1 of online supplementary material S1. The sample period begins from 2002 and ends to 2017 for two reasons: first, China’s farmland water development reached a new and relatively stable stage after the abolition of agricultural taxes in 2002; second, China has been vigorously pursuing agricultural water price reform since 2017, which may have had an impact on agricultural water use efficiency. Therefore, choosing data between 2002 and 2017 is beneficial to avoid other factors that are difficult to control with, such as policy shocks from interfering with the study.

### Agricultural water efficiency calculated using the DEA model

The input-oriented super-efficient Data Envelopment Analysis (DEA) model is used to calculate AWE, which measures the ability to create a certain output with the fewest feasible water inputs. The input-oriented model assesses the efficacy of factor inputs of the evaluated decision-making units (DMU) from an input perspective. It specifically examines the degree to which input factors can be reduced without compromising output levels. Irrigation water serves as an essential input in agricultural production. It is crucial to ensure agricultural output while striving for optimal efficiency in water usage. Consequently, minimizing the amount of irrigation water employed results in increased agricultural water use efficiency.

1 $${AWE}_{i,t}=\frac{{OAWI}_{i,t}}{{AAWI}_{i,t}}$$

where ${AWE}_{i,t}$ denotes province i at time t in terms of agricultural water efficiency. ${AAWI}_{i,t}$ is the actual agricultural water input of province i at time t, ${OAWI}_{i,t}$ is the optimal agricultural water input of province i at time t. The standard form of the DEA model yields a linear programming approach to achieve the least amount of inputs while guaranteeing outputs under certain constraints as follows:

2 $$\begin{gathered} \min \left( {\theta - \varepsilon \left( {\mathop \sum \limits_{i = 1}^{m} s_{i}^{ - } + \mathop \sum \limits_{j = 1}^{q} s_{j}^{ + } } \right)} \right) \hfill \\ {\text{s}}.{\text{t}}.\left\{ {\begin{array}{*{20}c} {\begin{array}{*{20}c} { \mathop \sum \limits_{{\begin{array}{*{20}c} {k = 1} \\ {k \ne j} \\ \end{array} }}^{n} \lambda_{k} x_{ik} + s_{i}^{ - } = \theta x_{i}\quad i = 1,2, \cdot\cdot\cdot, m} \\ { \mathop \sum \limits_{{\begin{array}{*{20}c} {k = 1} \\ {k \ne j} \\ \end{array} }}^{n} \lambda_{k} y_{jk} - s_{j}^{ + } = y_{j}\quad j = 1,2, \cdot\cdot\cdot, q } \\{\lambda_{k} \ge 0,\quad k = 1, \ldots , n } \\ \end{array} } \\ {s_{i}^{ - } \ge 0, s_{j}^{ + } \ge 0 } \\ \end{array} } \right. \hfill \\ \end{gathered}$$

For the *k* th Decision-Making Units (DMU), ${x}_{ik}$ denote the *i* th input indicator, ${y}_{jk}$ represent the *j* th output indicator. and ${s}_{i}^{-}$ and ${s}_{j}^{+}$ are input and output slack variables, respectively. ${\lambda }_{k}$ denote the weight coefficient. θ is the comprehensive production efficiency. When AWE is calculated, θ is obtained.

Equation (1) defines the way we use to calculate water use efficiency, and Eq. (2) defines linear programming inequalities which solve for the optimal agricultural water input for each province at each time period. Once the optimal agricultural water input is solved from a DEA solver software, agricultural water use efficiency can be calculated with Eq. (1).

**Figure 1:**
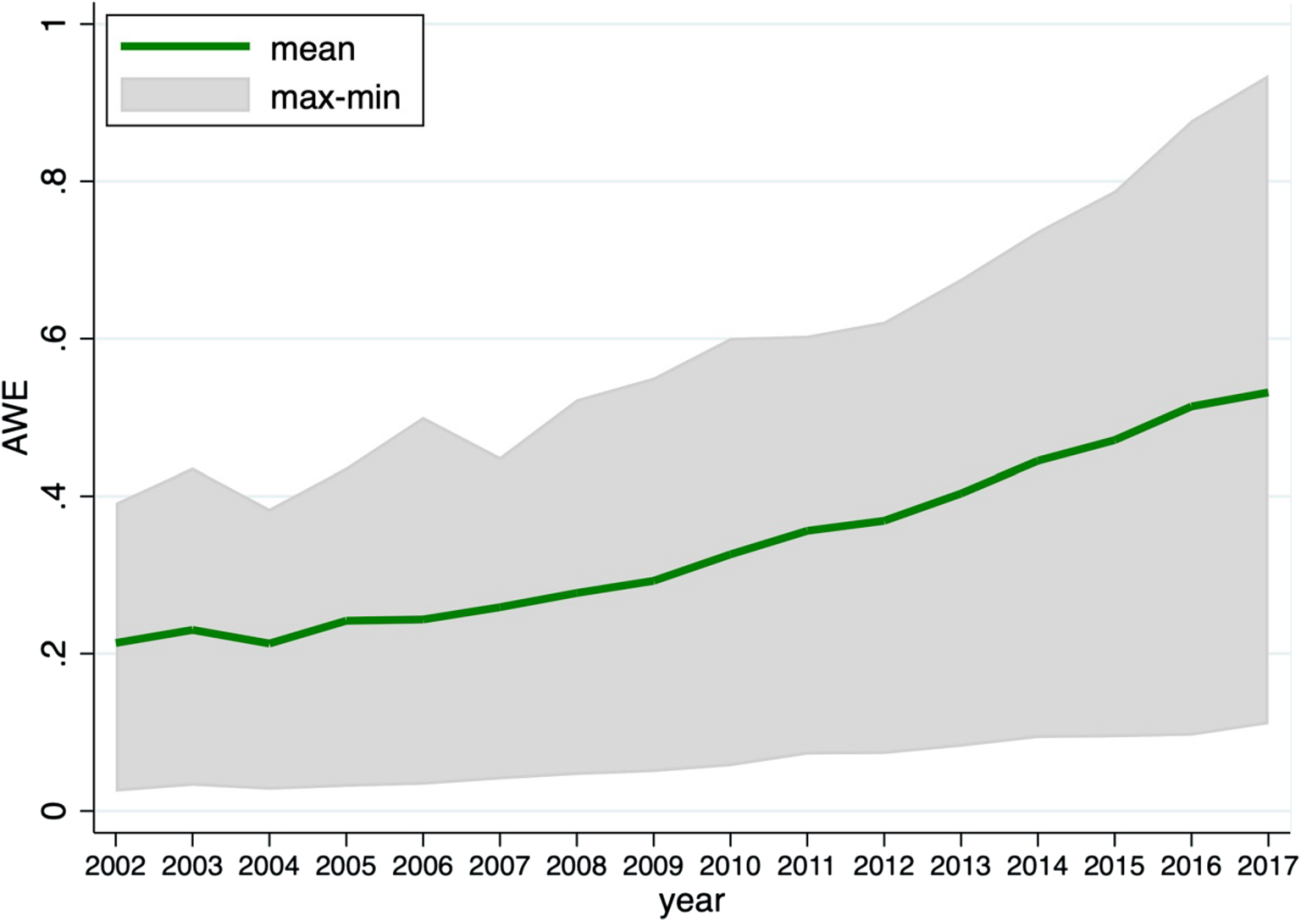
The time trend of China’s provincial level agricultural water efficiency.

The variables included in the super-efficient DEA model are as follows: the amount of pesticide sprayed to agricultural produce is used to calculate pesticide input. Fertilizer input is calculated using the quantity of nitrogen and phosphate fertilizer applied to agricultural produce. In agricultural production, diesel consumption is used as a surrogate for energy input. The total input of plastic film used in agricultural production is referred to as agricultural film. Land input is represented by the total area planted. Water input is proxied by total agricultural water usage. The yield value of the agricultural planting business is employed as a measure of production value. In addition, to avoid the influence of inflation, production estimates are deflated using 2002 as the base year. Figure 1 depicts the time trend of agricultural water use efficiency in China. The gray areas reflect the differences in agricultural water use efficiency between different regions of China.

### Energy poverty measures

The energy poverty indicators we have chosen reflect to some extent the local level of energy development. The main components are: individuals’ consumption of energy, which is the most basic level of energy development in a region. Individuals’ access to clean energy, which reflects the level of sophistication of a region’s energy mix. The energy-intensive furniture and equipment owned by people, which characterize the standard of living of individuals and the capacity of local energy facilities to supply energy. The level of energy management, a higher level of energy facilities requires investment in construction and capital, and careful management^15,16^. The energy poverty index is calculated using the Improved Entropy Method with methodology details presented in the online Supplementary material S1. Specific indicators for energy poverty measurement are shown in Table 1.

**Table 1:** Energy poverty indicators.

Indicator	Measurement
Accessibility of energy services (AES)	Electricity consumption per capita
	Natural gas use per capita
	Ratio of city dwellers who use natural gas
	Natural gas supply per capita in metropolitan regions
Cleanness of energy consumption (CEC)	Percentage of non-thermal power generation
	Biogas output per home in rural areas
Affordability and efficiency of energy (AEE)	Percentage of urban residences with air conditioning
	Per-hundred-households-owning-a-refrigerator
	Smoke exhaust ventilator ownership per hundred rural households
	Percentage of rural residences with smoke exhaust ventilators
	Per capita solar water heater coverage area in rural regions
	Per capita sulphur dioxide in waste gas from the residential sector
	Residential sector smoke and dust emissions per capita in waste gas
Management completeness for energy (MCE)	The number of rural energy management organisations per million persons
	In rural energy promotion organisations, the average number of staff is
	The per capita energy investment of rural inhabitants
	State-owned electricity, steam, and hot water generating and supply investment per capita

These indicators refer to Zhao et al.^16^.

### Econometric model

The influence of China’s EP on AWE is investigated in this study. Therefore, AWE is the dependent variable and EP is the independent variable. This research uses dynamic panel techniques to investigate the potential lag effects of AWE; the econometric model is constructed as follows:

3 $$Ln{AWE}_{it}=\alpha +{\beta }_{0}Ln{AWE}_{i,t-1}+{\beta }_{1}{EP}_{it}+{\beta }_{2}{X}_{it}+{\varepsilon }_{it}$$

where *i* signifies the analysis province and *t* denotes the year that the variable relates to. ${\varepsilon }_{it}$ denotes the random disturbance term with an independent and identical distribution. *α* stands for the intercept term. The estimated coefficients are denoted by ${\beta }_{i}$ (i ≥ 1). AWE stands for agricultural water efficiency. EP stands for energy poverty. X represents a vector that contains a collection of control variables, mainly consisting of the degree of water-saving irrigation (SAVE), education in rural areas (EDU), grain size per capital (GSC), water resource adequacy (WRA), and urbanization (URB).

Section 3.1 calculates agricultural water efficiency, whereas Sect. 3.2 calculates energy poverty. Furthermore, SAVE is calculated by dividing the water-saving irrigated area by the cultivated area. The fraction of the rural population with a high school diploma or above determines EDU. GSC is computed by dividing the area under cultivation by the number of farmers (thousand hectares/10 thousand persons). GSC represents the magnitude of agricultural output to some degree. WRA is derived by dividing regional water resources by crop area (100 million cubic metres per thousand hectares). URB refers to the percentage of permanent urban inhabitants in a region’s overall population. Table 2 offers descriptive data for these factors.

**Table 2:** Summary statistics of variables in the econometric model.

Var name	Indicator	Measure	Mean	SD	Min	Max
Agricultural water efficiency	*AWE*	Calculated from the DEA	0.025	0.935	0.337	0.180
Energy poverty	*EP*	According to the study of Zhao et al. (2021)	0.205	0.858	0.473	0.136
Degree of water-saving irrigation	*SAVE*	Proportion of water-saving irrigation area to total arable land area	0.036	1.000	0.253	0.219
Education level in rural areas	*EDU*	Percentage of population with high school or higher education	0.027	0.359	0.107	0.048
Grain size per capital	*GSC*	The total area sown divided by the number of people employed in agricultural production (thousand hectare / 10 thousand people)	2.478	24.692	5.917	3.030
Water resource adequacy	*WRA*	Measured by dividing the regional water resources by the area sown to crops (100 million m^3^/thousand hectare)	0.007	1.578	0.260	0.297
Urbanization	*URB*	Percentage of population with high school or higher education	0.269	1.295	0.539	0.153

## Results

### Cross-sectional dependency

Before conducting a meaningful econometric analysis, the existence of cross-sectional dependence in panel data must be evaluated. Unreliability and inconsistencies in empirical data analysis results are often attributed to a failure to consider cross-sectional independence^17^. As a result, the Friedman test^18^, the Breusch-Pagan LM test^19^, the Pesaran CD test^20^, and the Frees test^21^ are used in this research to examine cross-sectional dependence.

The results of the four tests of cross-sectional dependence are shown in Table 3. All statistics are significant at the 1% level. Therefore, we strongly disprove the null hypothesis, which states that there is no cross-sectional dependence. This indicates that the cross-sectional units were not independent in this research. Consequently, the cross-sectional dependence present in the data must be considered while performing the subsequent empirical investigation.

**Table 3:** Results of the cross-sectional dependence tests.

Test	Statistics	Prob
Pesaran CD test	10.948***	0.0000
Breusch–Pagan LM test	1971.300***	0.0000
Frees test	5.564***	0.0010
Friedman test	76.032***	0.0003

***p < 0.01.

### The impact of energy poverty on agricultural water efficiency

Because of the potential for endogeneity issues during the estimating process, appropriate econometric strategies are required in order to determine the effect that energy poverty has on AWE, which is the primary focus of this research. To begin, it is possible that erroneous measurements are what really create endogeneity. Especially prevalent in the social sciences is this problem since measurement of variables is hardly free from measurement error^22,23^. There is also the possibility of endogeneity due to omitted variable bias, which occurs when key variables are neglected in the model^22,23^. System generalized method of moments (SYS-GMM) has proven to be an effective statistical tool for handling issues of heterogeneity, endogeneity, and estimate bias^22,23^. SYS-GMM estimation method takes into account lagged values of the dependent variables to create internal instruments that resolve endogeneity problems^22,23^. The current levels of a phenomenon are best understood by looking at the values of lagged variables. The introduction of lags, according to Ullah's study, addresses the problem of "too many instruments."^22^.

System GMM (SYS-GMM) are employed as the benchmark method in this study. OLS, random effects model (RE), One-way fixed effects model (One-way FE) and difference GMM were used as robustness tests. Among the five estimation approaches, the sign and statistical significance of EP are basically consistent, indicating that the empirical results of this study are robust and reliable (Table 4).

**Table 4:** Estimation of EP-AWE nexus.

	Estimating static panel			Estimating dynamic panel	
	OLS	One-way FE	RE	DIF-GMM	SYS-GMM
*L.lnAWE*				0.912*** (0.033)	0.955*** (0.044)
*LnEP*	− 0.634*** (0.122)	− 0.148* (0.081)	− 0.162** (0.082)	− 0.186*** (0.020)	− 0.056** (0.028)
*LnSAVE*	− 0.244*** (0.039)	0.365*** (0.057)	0.267*** (0.054)	− 0.005 (0.017)	0.051** (0.020)
*LnEDU*	0.590*** (0.080)	0.703*** (0.047)	0.741*** (0.047)	0.061** (0.024)	0.031 (0.023)
*LnGSC*	− 0.341*** (0.075)	0.043 (0.084)	0.049 (0.081)	0.232*** (0.038)	0.179* (0.099)
*LnWRA*	− 0.063*** (0.024)	0.103*** (0.034)	0.106*** (0.032)	0.114*** (0.011)	0.078*** (0.014)
*LnURB*	0.321*** (0.121)	0.281*** (0.083)	0.251*** (0.084)	− 0.236*** (0.025)	− 0.186*** (0.035)
*Constant*	− 0.150 (0.307)	1.192*** (0.244)	1.082*** (0.256)	− 0.382*** (0.102)	− 0.147 (0.159)
Sargan test				0.316	0.999
Observations	480	480	480	420	450
*R-squared*	0.350	0.699	0.697		

***p < 0.01.; **p < 0.05; *p < 0.1; standard errors are in parentheses.

Table 4 shows the Sargan test results. According to Roodman (2009), the Sargan test of the two-step GMM estimates yields non-significant p-values, indicating that all instrumental variables used in this study have validity^24^.

With all five estimating methodologies, the influence of EP on AWE is negative and statistically significant, as shown in Table 3. For every one percent increase in energy poverty, AWE drops by around 0.056%. Improving AWE is critical to addressing water scarcity and ensuring food security. Energy poverty has a negative impact on the efficiency of water use in agriculture. Energy poverty reflects the level of development of energy infrastructure, such as the availability of electricity. Agricultural production systems are unable to use input factors efficiently due to inadequate energy supply. Many irrigation systems and water saving technologies require energy support and supply to operate. Although China has invested in the construction of many irrigation facilities, a stable supply of energy is required for the good operation of irrigation facilities. Many rural areas in China are still dominated by smallholder forms of production and lack the motivation and capacity to fund the management of irrigation systems and subsequent supporting energy facilities. This is an important reason why China has been making great efforts to develop the level of energy infrastructure in rural areas in recent years, and the Chinese government has made some achievements to achieve universal access to rural power electricity in 2020.

According to the SYS-GMM findings on control factors, EDU contributes to AWE. Better educated farmers are more likely to have sophisticated production expertise and irrigation methods that increase agricultural water use efficiency. AWE increases with the degree of SAVE, showing that water-saving irrigation is important for efficiency. GSC has a favorable impact on AWE. In China, a large number of smallholder farmers continue to dominate agricultural production. Increasing GSC minimizes the surplus of unproductive agricultural labor and increases AWE by promoting labor substitution through technology.

## Discussion

### Asymmetric analysis

**Figure 2:**
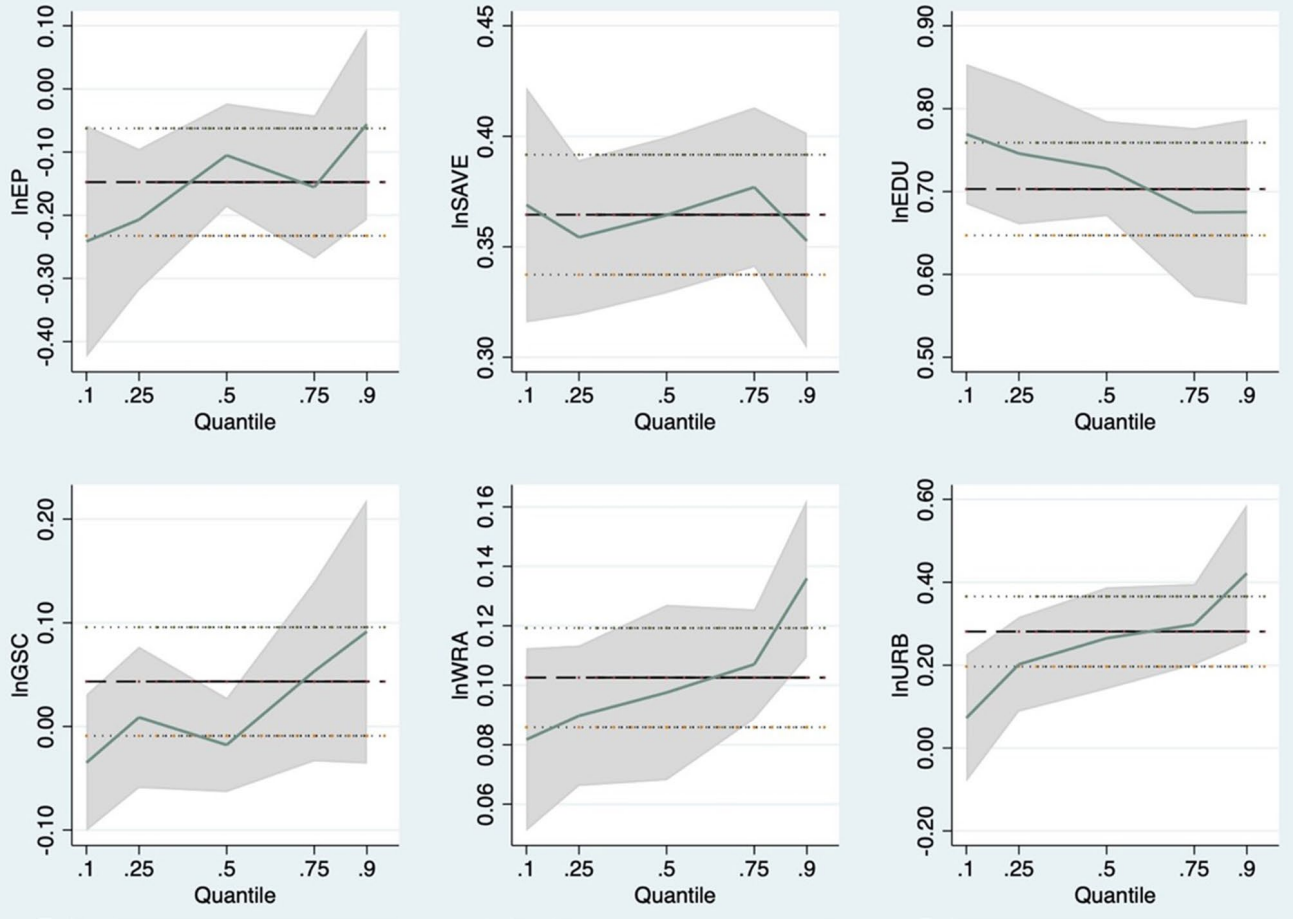
Change in panel quantile regression coefficients. The x-axis shows the conditional quantiles of AWE and the y-axis denotes the coefficient values of different variables. The coefficient values of a panel data model with a fixed effect are shown by the black line.

The 10th, 25th, 50th, 75th and 90th quartiles of the AWE were estimated to account for unobserved heterogeneity in order to quantify the asymmetry of the impact of energy poverty on agricultural water use efficiency. The main two-step panel quantile regression was used^25^. The estimated results are summarized in Table 5. Furthermore, at various quantile stages, Fig. 2 demonstrates the varied patterns of change in the parameters of affecting factors. EP and control factors affect AWE to different degrees at different quartile levels. When AWE is low, energy poverty has a stronger negative impact. At higher levels of AWE, the negative impact of energy poverty fades away. For the control variables, it is worth noting that as AWE increases, the role of urbanization level becomes more and more important, suggesting that higher levels of AWE need to be driven by regional economic development.

**Table 5:** Estimation of two-step panel quantile regression.

Dependent variable: *lnAWE*					
Independent variables	Quantiles				
	q10	q25	q50	q75	q90
*lnEP*	− 0.241*** (0.071)	− 0.207*** (0.058)	− 0.105*** (0.048)	− 0.155*** (0.051)	− 0.056 (0.056)
*lnSAVE*	0.369*** (0.015)	0.354*** (0.013)	0.364*** (0.019)	0.377*** (0.013)	0.353*** (0.023)
*lnEDU*	0.769*** (0.038)	0.746*** (0.035)	0.728*** (0.036)	0.675*** (0.042)	0.675*** (0.039)
*lnGSC*	− 0.035 (0.044)	0.009 (0.041)	− 0.018 (0.036)	0.053 (0.055)	0.092 (0.080)
*lnWRA*	0.082*** (0.010)	0.090*** (0.008)	0.098*** (0.008)	0.107*** (0.011)	0.136*** (0.011)
*lnURB*	0.073 (0.063)	0.202*** (0.065)	0.265*** (0.074)	0.298*** (0.047)	0.422*** (0.091)
*Constant*	1.030*** (0.178)	1.089*** (0.125)	1.356*** (0.159)	1.257*** (0.217)	1.492*** (0.181)

***p < 0.01; **p < 0.05; *p < 0.1; standard errors are in parentheses.

### Mechanism analysis

#### Potential mechanisms

The obvious possibility is that irrigation machinery cannot be used without energy^26,27^, so energy poverty may have reduced the number of irrigation machinery, thus affecting AWE.

SDG2 explicitly proposes to promote non-farm employment for small-scale food producers. Furthermore, in the study of rural China, non-farm work has always been an unavoidable issue^28,29^. Farm families’ human capital and cognitive capacity may be eroded as a result of energy poverty^30–32^, compromising their ability to work outside of the farm. Non-farm employment promotes farmers to invest more and master agricultural technology to improve agricultural water efficiency by improving agricultural income and cognitive ability^26,29,33–35^. Most importantly, non-agricultural employment can effectively address the issue that Chinese agricultural production is still dominated by a large number of scattered, small-scale food producers. It can promote land concentration and thus large-scale production, which clearly facilitates the adoption of community-based water-saving technologies.

The literature has not taken into account the impact of energy poverty on cropping structure adjustment, which may be detrimental to cash crop cultivation, such as the inability to provide warm barns to withstand the cold. Cash crops, on the other hand, may promote agricultural water efficiency. For starters, cash crops can boost farm income, allowing farmers to invest in and maintain water-saving irrigation systems. Second, cash crops frequently necessitate fine-grained management and good infrastructure, both of which aid in reducing water waste and increasing AWE.

In order to investigate the mechanism of how EP affects AWE, the quantity of water-saving irrigation equipment (WIM), non-farm work (NFW), and cropping structure adjustment (CSA) are employed as mediating factors in this model. The number of water-saving irrigation machines is referred to as WIM. NFW is proxied by the ratio of non-agricultural employment to total employment. The percentage of vegetable planted area to all crops seeded is used to calculate CSA. The following is the mediating effect model that was used to investigate the mechanism:

4 $$Ln{AWE}_{it}={\delta }_{1}Ln{EP}_{it}+{\beta }_{1}{X}_{it}+{\phi }_{it}$$

5 $$Ln{M}_{it}={\delta }_{2}Ln{EP}_{it}+{\beta }_{2}{X}_{it}+{\mu }_{it}$$

6 $$Ln{AWE}_{it}={\delta }_{3}Ln{EP}_{it}+{\delta }_{4}Ln{M}_{it}+{\beta }_{3}{X}_{it}+{\gamma }_{it}$$

where *i* signifies the analyses’ cross-sectional unit. And *t* stands for time periods, while AWE stands for agricultural water efficiency at the provincial level. *EP* stands for energy poverty. *X* presents a set of control factors to the user. WIM, OFM, and CSA are among the mediators represented by *M*. EP’s entire impacts on AWE are represented by ${\delta }_{1}$. ${\delta }_{3}$ denotes the direct influence of EP on AWE. In addition, ${\delta }_{2}\cdot {\delta }_{4}$ is the indirect impact.

#### Results of the mediation effect analysis

Table 6 shows the estimated results of the mediation influence mechanism. First and foremost, the overall effects of EP on AWE (${\delta }_{1}$) are statistically significant, as shown in Column (1) of Table 6, and the elasticity is − 0.504. Second, Table 6’s Columns (2)–(4) demonstrate that ${\delta }_{2}$ of the mediators, WIM, NFW, and CSA, are both statistically significant at the 1% level, with elasticities of − 2.002, − 0.274, and − 0.501, respectively. EP had a negative influence on the WIM, NFW, and CSA, according to these data. Third, the coefficients of WIM, NFW, and CSA are all statistically significant, with elasticities of 0.087, 0.346, and 0.116, respectively, as shown in Columns (5)–(7). WIM, NFW, and CSA are all substantial contributors to AWE, as seen by this. The EP coefficient, on the other hand, falls, indicating that the mediators are partly mediating.

**Table 6:** Mechanism analysis results.

Variables	(1)	(2)	(3)	(4)	(5)	(6)	(7)
	*lnAWE*	*lnWIM*	*lnNFW*	*lnCSA*	*lnAWE*	*lnAWE*	*lnAWE*
*lnWIM*					0.087*** (0.011)		
*lnNFW*						0.346*** (0.114)	
*lnCSA*							0.116*** (0.030)
*lnEP*	− 0.504*** (0.063)	− 2.002*** (0.174)	− 0.274*** (0.026)	− 0.501*** (0.077)	− 0.311*** (0.075)	− 0.411*** (0.066)	− 0.379*** (0.073)
*lnSAVE*	− 0.223*** (0.026)	− 0.158*** (0.051)	0.048*** (0.007)	0.137*** (0.018)	− 0.177*** (0.026)	− 0.247*** (0.027)	− 0.254*** (0.028)
*lnEDU*	0.635*** (0.044)	0.545*** (0.109)	0.098*** (0.016)	0.409*** (0.041)	0.513*** (0.050)	0.584*** (0.044)	0.620*** (0.050)
*lnGSC*	− 0.233*** (0.049)	1.358*** (0.095)	0.093*** (0.014)	− 1.105*** (0.046)	− 0.359*** (0.051)	− 0.269*** (0.049)	− 0.186*** (0.057)
*lnWRA*	− 0.074*** (0.016)	− 0.172*** (0.042)	0.005 (0.005)	0.013 (0.012)	− 0.066*** (0.018)	− 0.060*** (0.016)	− 0.076*** (0.019)
*lnURB*	0.075 (0.068)	− 1.667*** (0.201)	0.451*** (0.024)	0.229*** (0.070)	0.406*** (0.082)	− 0.023 (0.074)	0.065 (0.080)
*Constant*	− 0.210 (0.158)	5.456*** (0.414)	− 0.283*** (0.061)	0.674*** (0.179)	− 0.686*** (0.175)	− 0.097 (0.155)	− 0.038 (0.174)
*Observations*	480	480	480	480	480	480	480
*Number of id*	30	30	30	30	30	30	30

***p < 0.01; **p < 0.05; *p < 0.1; standard errors are in parentheses.

Energy poverty has an immediate and negative impact on AWE, as well as three intermediary channels. Energy poverty reduces the availability of water-saving irrigation machinery, lowering AWE. Energy poverty discourages non-agricultural employment. The lack of non-farm employment reduces farm households’ ability to invest in and maintain irrigation facilities. As a result, non-agricultural employment is a critical channel for EP to reduce AWE. Farmers are discouraged from growing high-value cash crops like vegetables due to energy shortages. Energy poverty makes it difficult to build facilities for high-value cash crops that require energy inputs, such as greenhouses that can stay warm during cold winters. Farmers are unable to increase their income due to their inability to grow high-value cash crops, and they lack the incentive or resources to support more advanced irrigation management facilities.

## Conclusions

The study's primary discoveries can be summarized as follows: (1) The efficiency of agricultural water utilization in China experiences a detrimental impact due to the presence of energy poverty. More precisely, a 1% rise in energy poverty is associated with a 0.056% decrease in agricultural water efficiency. As a result, the condition of energy poverty exerts an adverse influence on the efficiency of agricultural water utilization. (2) Based on the findings of an asymmetrical study, an enhancement in agricultural water efficiency leads to a diminishing detrimental effects of energy poverty while the beneficial effects of urbanization become more significant. (3) According to the findings of mechanism analysis, energy poverty diminishes agricultural water efficiency by limiting the availability of irrigation water-saving machinery and hinders samllholder farmers from accessing off-farm employment opportunities. Furthermore, energy poverty adversely affects agricultural water efficiency by altering the cropping structure.

The aforementioned research findings carry several important policy implications. Firstly, it is evident from empirical results that the reduction of energy poverty is crucial in enhancing irrigation efficiency. Hence, it is important for the government to ascertain the underlying factors contributing to energy poverty among small-scale food producers in order to facilitate the implementation of tailored solutions. Energy poverty can potentially impact small-scale food producers, primarily as a consequence of inadequate infrastructure or limited household income. Policies aimed at augmenting infrastructure investment are implemented to cater to the needs of small-scale food producers who face challenges due to inadequate energy infrastructure. Off-site relocation can be employed as a strategy to facilitate the migration of individuals facing energy poverty towards regions characterized by improved infrastructure. In light of inadequate household income, it is recommended that the government extend energy subsidies and agricultural production subsidies to small-scale food producers. Special consideration should be granted to small-scale food producers due to the government's implementation of a clean energy transition, which has led to an increase in energy costs.

Furthermore, as agricultural water usage becomes more efficient, the negative impacts of energy poverty diminish while the positive effects of expanded production become more pronounced. In regions with low agricultural water use efficiency, it is imperative to prioritize the elimination of energy poverty and the enhancement of energy infrastructure as means to enable effective irrigation practices. Further improvements in agricultural water use efficiency at advanced stages can be achieved through the utilization of cutting-edge technology and financial assistance provided by the local economic development initiatives.

Thirdly, studies examining potential impact pathways indicate that water-saving irrigation equipment, non-farm employment, and cropping structure are three significant mediating factors. The correlation between energy poverty and agricultural water efficiency (AWE) is evident and understandable due to the constraining effect on irrigation equipment accessibility. Moreover, energy poverty impedes the progress of non-farm employment. Energy poverty has a detrimental impact on the human capital of farmers, thereby constraining their occupational options to non-agricultural sectors that necessitate elevated levels of skill proficiency. Meanwhile, the presence of energy poverty in urban areas hinders their capacity to accommodate and utilize rural labor. Enhancing the educational attainment in rural areas and providing vocational training to underdeveloped regions can contribute to the augmentation of farmers' human capital and the promotion of non-agricultural employment. Consequently, this can help alleviate the adverse effects of poverty on irrigation efficiency. The eradication of energy poverty and the promotion of rural non-farm employment have positive implications for enhancing agricultural water use efficiency. Furthermore, the presence of energy poverty acts as a hindrance to the cultivation of lucrative crops. The cultivation of lucrative crops has the potential to enhance agricultural water efficiency (AWE) by augmenting farmers' income and necessitating more meticulous resource management. This, in turn, incentivizes farmers to allocate greater investments towards the improvement of irrigation systems. In the context of enhancing energy infrastructure, it is advisable for the government to consider providing subsidies to farmers, offering training programs to enhance their skills, promoting the adoption of advanced management techniques for cultivating high-value lucrative crops, augmenting farmers' income, and enhancing irrigation facilities.

This study is subject to several limitations. First, the lack of detailed data restricts the ability to make more nuanced conclusions, particularly regarding the inclusion of temporal data at a quarterly or monthly level. Second, the study sample is limited to the provincial level, overlooking potential variations within cities or counties of the same province. Third, this study only focuses on a macro water use perspective and doesn’t differentiate water use efficiency by crop types. Different types of crops may have heterogeneous energy and water requirements, therefore disaggregated water use data by crop types can yield more accurate statistical resutls. Future studies could address these limitations by incorporating novel sources of refined data, such as satellite remote sensing data, high-frequency energy consumption data, and disaggregated water use data by specific crop types.

### Supplementary Information

Supplementary Information.

## Data Availability

The datasets used and/or analysed during the current study available from the corresponding author on reasonable request.
