# Supplementary material for: Influence of energy poverty on agricultural water efficiency using a panel data study in China
Source: Sci Rep. 2024 Jan 24;14:2064. doi: 10.1038/s41598-023-50971-y (PMC10808184; doi:10.1038/s41598-023-50971-y)
Supplement: Supplementary file 1 — Supplementary Information. [file 41598_2023_50971_MOESM1_ESM.docx]

**Supplementary materials**

| Variable | Data source | Role in analysis |
| --- | --- | --- |
| *AWE* | China National Bureau of Statistics  China Rural Statistics Yearbook | Dependent variable：  Measurement of a unit's ability to maximize agricultural output with minimal water inputs |
| *EP* | Zhao et al. (2021)  China Statistical Yearbook  China Energy Statistics Yearbook | Core independent variables：  Comprehensive measurement of energy poverty in a region |
| *SAVE* | China Rural Statistics Yearbook | Control variable：  Controlling the impact of water conservation |
| *EDU* | China Rural Statistics Yearbook | Control variable：  Controlling the impact of human capacity |
| *GSC* | China Rural Statistics Yearbook | Control variable：  Controlling the impact of the scale of agricultural production |
| *WRA* | China National Bureau of Statistics | Control variable：  Control of impacts due to regional water resource endowments |
| *URB* | China Statistical Yearbook  China National Bureau of Statistics | Control variable:  Control of the impact of the level of socio-economic development of the region |
| *WIM* | China Rural Statistics Yearbook | Mediation variable:  Measuring Irrigation Mechanization Levels, EP-AWE's Intermediate Channel |
| *NFW* | China Statistical Yearbook  China Rural Statistics Yearbook | Mediation variable:  Measuring labor migration from the agricultural sector to the non-agricultural sector, EP-AWE's Intermediate Channel |
| *CSA* | China Rural Statistics Yearbook | Mediation variable:  Measuring changes in crop structure (area sown to cash crops/area sown to food crops), EP-AWE's Intermediate Channel |

Table A1. Data source and variables used in the statistical analyses.

Note ：The estimation technique for the mediation model uses feasible generalized least squares to fit panel data linear models. Using the 'xtgls' command in STATA software.

Description of the Improved Entropy Method

For positive measurements, the normalization is expressed as Eq.1 . For negative measurements, the normalization is expressed as Eq.2 .

| $x_{ij}^{'}=\frac{x_{ij}-min(x_{1j} ,..., x_{nj})}{max(x_{1j} ,..., x_{nj})-min(x_{1j} ,..., x_{nj})}$ | (1) |
| --- | --- |
| $x_{ij}^{'}=\frac{max(x_{1j} ,..., x_{nj})-x_{ij}}{max(x_{1j} ,..., x_{nj})-min(x_{1j} ,..., x_{nj})}$ | (2) |

The following formula (Eq.3) is used in this study to compute the ratio of the value of the j-th measurement of the i-th province to the value of the j-th measurement of all provinces:

| $p_{ij}=\frac{x_{ij}^{'}}{\sum_{i=1}^{n} x_{ij}^{'}}$ | (3) |
| --- | --- |

The entropy value of the j-th measurement is then calculated as follows (Eq4):

| $e_{j}=-k\sum_{i=1}^{n} p_{ij}Ln(p_{ij})$ | (4) |
| --- | --- |

where $k=1Ln(n)>0;e_{j}\geq0$.

The following (Eq.5) is how this study calculates the information entropy redundancy:

| $d_{j}=1-e_{j}$ | (5) |
| --- | --- |

The weight of each measurement is calculated in the following manner (Eq.6):

| $w_{j}=\frac{d_{j}}{\sum_{j=1}^{m} d_{j}}$ | (6) |
| --- | --- |

The calculation of the energy poverty involves a comprehensive index (EP), which can be determined through the following steps (Eq.7):

| ${EP}_{i}=\sum_{j=1}^{n} w_{j}\cdot x_{ij}^{'}$ | (7) |
| --- | --- |

The specific indicators corresponding to the formula are the variables in Table 1. The energy poverty index can be calculated by bringing them into the above formula and following the steps. This study obtains the comprehensive energy poverty indexes (EP) based on the preceding calculation processes.
